# Approaches to Peripheral Artery Disease in Diabetes: Are There Any Differences?

**DOI:** 10.3390/ijerph19169801

**Published:** 2022-08-09

**Authors:** Alexandru Achim, Agata Stanek, Călin Homorodean, Mihail Spinu, Horea Laurenţiu Onea, Leontin Lazăr, Mădălin Marc, Zoltán Ruzsa, Dan Mircea Olinic

**Affiliations:** 1 Medical 1 Clinic, Department of Interventional Cardiology, University of Medicine and Pharmacy “Iuliu Hatieganu”, 400000 Cluj-Napoca, Romania; 2 ”Niculae Stancioiu” Heart Institute, University of Medicine and Pharmacy “Iuliu Hatieganu”, 400000 Cluj-Napoca, Romania; 3 Klinik für Kardiologie, Medizinische Universitätsklinik, Kantonsspital Baselland, 4410 Liestal, Switzerland; 4 Internal Medicine Department, Division of Invasive Cardiology, University of Szeged, 6720 Szeged, Hungary; 5 Department of Internal Medicine, Angiology and Physical Medicine, Faculty of Medical Sciences in Zabrze, Medical University of Silesia, Batorego 15 St., 41-902 Bytom, Poland

**Keywords:** peripheral artery disease, diabetes mellitus, revascularization, guidelines

## Abstract

Peripheral artery disease (PAD) increases the risk of diabetes, while diabetes increases the risk of PAD, and certain symptoms in each disease increase the risk of contracting the other. This review aims to shed light on this harmful interplay between the two disorders, with an emphasis on the phenotype of a patient with both diabetes and PAD, and whether treatment should be individualized in this high-risk population. In addition, current guideline recommendations for the treatment of PAD were analyzed, in an attempt to establish the differences and evidence gaps across a population suffering from these two interconnected disorders.

## 1. Introduction

The phenotypic manifestations of atherosclerosis vary in each individual and throughout the body; it is not fully understood why plaque formation has such a heterogeneous distribution, although different arterial systems are correlated [1,2,3]. Moreover, risk factors (such as diabetes mellitus, hypertension, smoking, etc.) undoubtedly aggravate atherogenesis and cardiovascular mortality through a dual risk: firstly, the intrinsic risk of the underlying disease; secondly, it increases the risk of atherosclerosis in various target organs. In other words, the co-prevalence of two atherogenic conditions is not simply an association due to shared risk factors; one may in fact play a fundamental role in the pathophysiology of the other and vice versa. We know that there is a bidirectional relationship between the risk of cardiovascular disease and systemic diseases, such as diabetes mellitus or primary hypertension. Thus, the complex relationship between atherogenesis and risk factors intersects at several levels, and perhaps the severity of the patient’s clinical manifestations best demonstrates this harmful interplay [4,5].

An evocative example would be peripheral artery disease (PAD), which is a direct macrovascular disorder of diabetes mellitus (DM). PAD raises the risk of DM, and DM raises the risk of PAD. Each 1% increase in hemoglobin A1c (HbA1c) is associated with a nearly 30% increase in the risk of developing PAD during the follow-up period. PAD also manifests earlier in diabetics and progresses more rapidly to critical limb ischemia. In addition, a diabetic patient with PAD has a 14.2% increased risk of major cardiovascular events when HbA1c increases by 1% [6,7]. This deleterious interaction is self-evident, although the relationship between diabetes and vasculopathy has not been fully elucidated, despite considerable research efforts in molecular, animal, and translational models. Currently, the consequences of peripheral ischemia are thought to result from the interaction between hemodynamic, neurohumoral, and metabolic factors, leading to endothelial dysfunction. However, numerous fundamental questions remain unanswered. For instance, the phenomenon of the regression of microvascular disease following strict glycemic control is not observed in the larger vessels [5]. This situation highlights the need for an aggressive multidisciplinary approach to limb salvage in the diabetic population. This review seeks to shed light on the phenotype of the diabetic patient with PAD and to discuss whether treatment should be individualized for this subpopulation.

## 2. Is PAD Different in Patients with DM?

More than 170 million people worldwide have DM, and this disease burden is expected to increase to nearly 370 million people in the next 10 years [8]. In 2010, more than 200 million people around the world were living with PAD [9]. The Framingham Heart Study [10] found that 20% of PAD symptomatic patients had DM. However, this prevalence is probably greatly underestimated, considering that most people with PAD are asymptomatic rather than symptomatic. Indeed, more than half of the patients with PAD have been found to be asymptomatic or have unusual symptoms, while one-third have claudication, and the remainder have more severe forms of the illness [11]. A patient with diabetes and PAD is more likely to develop an ischemic ulcer or gangrene than a patient without diabetes [11]. The pathophysiology of PAD in the diabetic population is similar to that of the non-diabetic population but is exacerbated by the presence of concomitant DM. Apart from the atherogenic consequences of diabetes-related dyslipidemia (elevated triglycerides, low HDL cholesterol levels, and small/dense LDL particles), several clinical and experimental investigations have shown that high insulin levels precede the development of arterial damage [12,13]. The underlying metabolic disturbances in diabetes promote vascular inflammation, endothelial dysfunction, vasoconstriction, platelet activation, and thrombotic risk, all of which contribute to the etiology of PAD in diabetic individuals. Clinically, the increase in overall risk caused by DM is driven by the accelerated progression of preexisting atherosclerosis into clinical cardiovascular events [14,15].

### 2.1. The Biological Level

**Figure 1:**
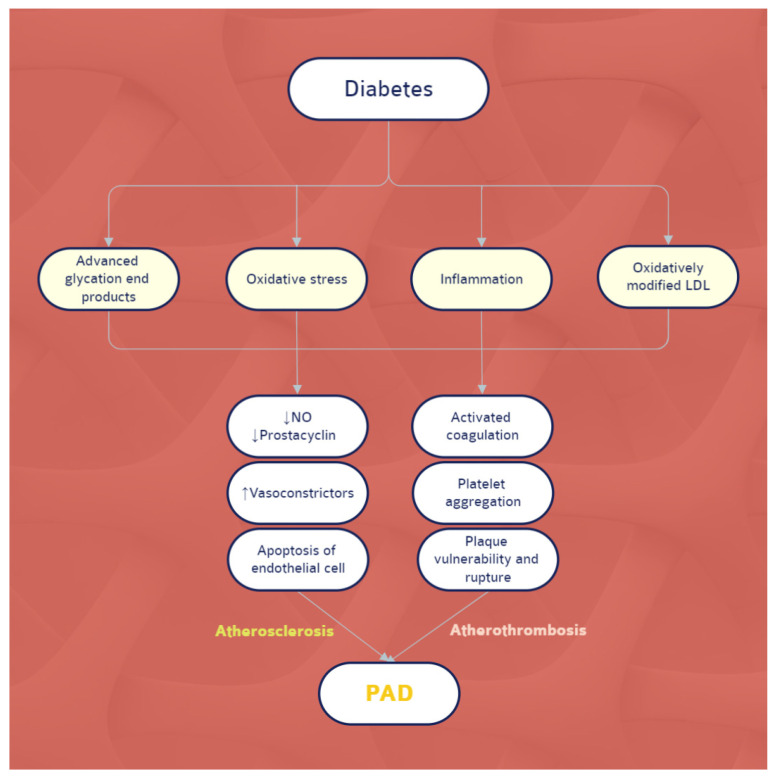
The pathophysiological hallmarks of PAD in DM. Abbreviations: NO, nitric oxide; LDL, low-density lipoprotein; PAD, peripheral artery disease.

The profound effects of DM on the atherothrombotic milieu of the peripheral vasculature extend at multiple levels. First, a proinflammatory condition, as measured by elevated levels of the C-reactive protein (CRP), is found in both PAD [16] and DM [17]. CRP has procoagulant properties as it stimulates the expression of tissue factors [18]. Diabetes-related hyperglycemia also causes an increase in mitochondrial reactive oxygen species via the protein kinase C (PKC) pathway, which acts as a causal link between high blood glucose and major adverse vascular outcomes [19]. Upon activation, PKC causes structural and functional changes in arteries, including cellular permeability, inflammation, angiogenesis, cell proliferation, expansion of the extracellular matrix, and apoptosis [20]. Second, endothelial dysfunction is very common in individuals who have both diabetes and PAD. Diabetic hyperglycemia causes an imbalance between nitric oxide bioavailability and reactive oxygen species buildup, resulting in poor vascular function [19]. Reactive oxygen species also increase the advanced glycation end products, which cause endothelial dysfunction and disrupt vascular homeostasis [19]. Third, increased protein kinase C activity causes increased endothelin-1 synthesis, leading to greater vasoconstriction and platelet aggregation [20]. Furthermore, protein kinase C activation modifies the nitric oxide signaling process and stimulates vasoconstriction [21]. Finally, both insulin resistance and hyperglycemia contribute to the development of a prothrombotic state characterized by increased platelet activation and coagulation [21,22]. Several pathophysiologic mechanisms contribute to platelet dysfunction and hyperreactivity in DM [19], many of which may also play a role in the pathology of PAD [23]. Hyperglycemia damages platelet-calcium homeostasis and stimulates the production of factors that increase platelet aggregability [24]. Another pathway could be via the vesicles released into the bloodstream by various cell types during apoptosis or activation. These are elevated in DM and can independently predict cardiovascular events in individuals with stable coronary artery disease [25]. Once they reach the circulation, these vesicles activate procoagulant mechanisms in the endothelial cells of patients with DM and promote thrombus formation at the site of injury [26]. Increased platelet-derived vesicles are also associated with PAD and may indicate a common etiology for PAD risk in diabetic individuals [23]. The aforementioned pathophysiological mechanisms are briefly illustrated in Figure 1.

### 2.2. The Clinical Level

The clinical symptoms of PAD consist initially of claudication and pain at rest and, in more advanced stages, of ulceration and gangrene. These symptoms are usually caused by progressive luminal constriction (stenosis/occlusion), although the thrombosis or embolism of unstable vulnerable atherosclerotic plaques or material may also occur. The impact of PAD can be assessed by its progression, the prevalence of symptoms, and the excess cardiovascular events linked with systemic atherosclerosis. Consequently, DM is a major risk factor for PAD, surpassed only by cigarette smoking in terms of the magnitude of increased risk [27]. DM is present in approximately 30% of patients with claudication and 50% of patients with critical limb ischemia [28]. Pain at rest and claudication may go unnoticed in patients with diabetes because of coexisting sensory neuropathy. Amputations are common in diabetic patients because of associated refractory ulcers, comorbidities, and end-organ injury [14,29]. It is important to note that diabetes is more closely related to femoral-popliteal and tibial (below the knee) PAD, whereas other risk factors (such as smoking and hypertension) are linked to more proximal disease in the aorto-iliofemoral system. A multisegmental presentation with long, calcified stenoses/occlusions of the lower leg arteries and inadequate collateral formation is representative. The risk of developing the complications of DM increases with increasing concentrations of hyperglycemia [5]. HbA1c levels but not DM duration predicts a more severe PAD [30,31]; however, interestingly, the rate of increase in the risk of microvascular disease with exposure to glycemia over time is greater than that for macrovascular disease [32]. The first evidence of this phenomenon was reported in a study by Lorenzi et al. in the 1980s, in which the authors showed that the microvascular changes induced by hyperglycemia persisted after the restoration of normoglycemia [33]. In the UKPDS trial, the relative risk of microvascular disease with hyperglycemia was greater than that of myocardial infarction, demonstrating the decisive role of hyperglycemia in the etiology of small vessel disease; this result may explain the greater rate of microvascular complications seen in populations with less satisfactory glycemic control [34]. Moreover, patients with additional comorbidities, such as chronic obstructive pulmonary disease, are more likely to have more advanced and multilayered atherosclerotic lesions [35]. Thus, the multi-faceted pathology of DM in microvascular and macrovascular districts can be observed.

The contemporary approach to these infrapopliteal lesions has been improved by the dual vascular approach [36] and the use of drug-eluting balloons [37]. Recent studies suggest an additional benefit in treating infrapopliteal lesions in patients with diabetes and chronic limb-threatening ischemia with paclitaxel-eluting balloons [38]. DM may adversely affect peripheral stent restenosis and target lesion revascularization [39,40]. It has also been shown that adults with PAD and concomitant DM exhibited worse lower extremity function than those who were only diagnosed with PAD [37]. Impaired lower extremity function is an important predictor of future incapacity, including the loss of mobility and placement in a rehabilitation center. As a result, a patient with DM and PAD demonstrates a high-risk clinical profile that should be evaluated for comorbidities such as neuropathy, foot ulceration, or concurrent coronary artery disease, which could contribute to incapacity and disability [37]. A diabetic patient is polyarterial, meaning that the deleterious processes of glycosylation do not spare the coronary, carotid, or cerebral arteries [41,42].

## 3. Diagnosis

A comprehensive medical history and physical examination are essential for determining the existence of PAD in patients with DM. During a physical examination, blood pressure should be measured, and the peripheral pulses should be palpated at the femoral, popliteal, and pedal vessels. It is beneficial to have information on the symptom onset and duration, pain characteristics, and any mitigating factors. However, leg pain and functional impairment may also be secondary to diabetic neuropathy [43]. The Fontaine scale can be used to classify the clinical stages of symptomatic PAD. Fontaine stage-I patients have PAD but are asymptomatic; patients at stages IIa and IIb have mild and moderate-to-severe intermittent claudication, respectively; Fontaine stage-III patients have symptoms at rest, and Fontaine stage-IV patients have significant tissue loss (ulcers or gangrene) [44].

The ankle-brachial index (ABI) is used to confirm the diagnosis of PAD in individuals after collating an adequate history and physical examination. The ABI is calculated by dividing the higher systolic blood pressure of the right or left arm by the higher systolic blood pressure of the posterior tibial or dorsalis pedis in each leg. An ABI of 0.90 is sensitive and is specific for arterial stenosis, and is also diagnostic for PAD, whereas further testing may be required in diabetic individuals [45]. An ABI after exercise testing may provide additional information for people with the relevant symptoms and a normal ABI. The ABI is limited in mediocalcinosis, which typically occurs in patients with DM. Diffuse calcification of the arterial wall may cause the tibial artery to become incompressible, limiting systolic pressure measurement at that location despite inflating the blood pressure cuff up to 250 mmHg; in consequence, stenotic lesions cannot be detected by the ABI. In this situation, other noninvasive tests, such as the measurement of the toe-brachial index (TBI) or Doppler waveform analysis may reveal vascular occlusive disease, despite a falsely elevated ABI [46]. The measurement of TBI is important in this context because digital vessels rarely calcify and can provide an accurate assessment of vascular disease in the presence of calcification. As with coronary arteries [47], the peripheral fractional flow reserve can be measured invasively via stenosis with a pressure wire; although there have been several promising attempts, technical drawbacks, such as unknown cut-off values and unknown optimal papaverine/adenosine doses, have made this measurement unpopular in terms of peripheral vasculature [48].

Invasive angiography remains the gold standard for diagnosis, but over the past decade, there has been an increasing reliance on noninvasive imaging studies to diagnose PAD. Ultrasonic duplex scanning can directly visualize the vessels, providing information about the thickness of the arterial wall, the degree of flow turbulence, and changes in blood flow velocity. With the introduction of magnetic resonance (MR) angiography and computed tomographic (CT) angiography, non-invasive imaging is now a reality. Contrast-enhanced magnetic resonance angiography produces images that are comparable to conventional angiography. More recently, the development of CT angiography has dramatically improved image quality and expanded the applications of non-invasive angiography [46]. Between 2002 and 2013, a study found that among radiologists, MR and CT angiography almost replaced invasive angiography in the diagnosis of PAD. However, the phenomenon was not uniform, as the use of invasive angiography increased sharply among cardiologists and surgeons despite there being available noninvasive alternatives [49]. This figure demonstrates the need for unanimity among specialties and for the introduction of these tests in consensuses and guidelines. Moreover, volumetric CT perfusion has recently shown promising results in the assessment of PAD before and after revascularization [50,51], while a new MR perfusion protocol reliably differentiated patients with PAD from healthy controls [52]. The laser Doppler blood flowmeter is another useful noninvasive tool for detecting PAD at an early stage by recording skin perfusion deterioration [53].

## 4. Management: Differences between Guidelines

**Figure 2:**
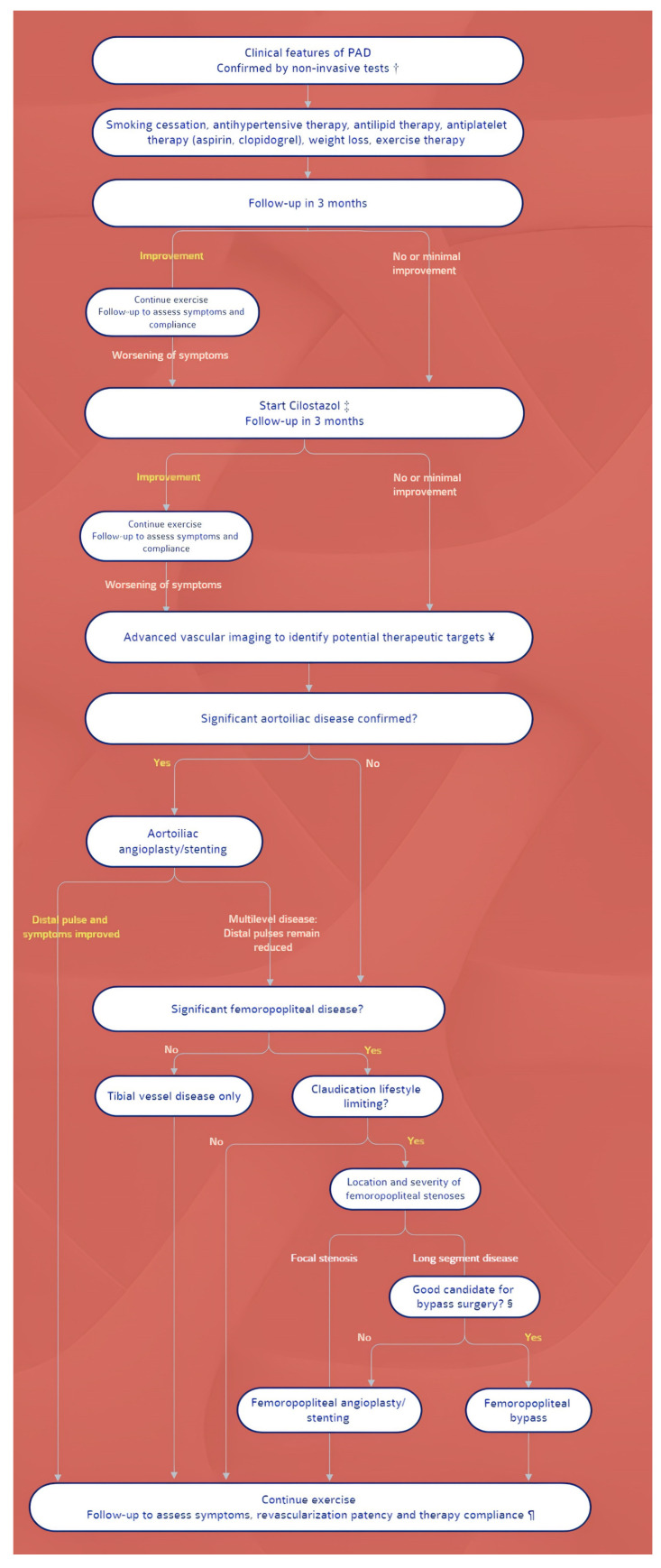
Algorithm for the management of PAD. Symbols: † Noninvasive studies include ankle-brachial pressure index, pulse volume recordings, partitioned pressures, and exercise testing. ‡ Cilostazol is recommended for patients with moderate-to-severe claudication. For patients with contraindications or for patients who do not tolerate cilostazol, pentoxifylline is an alternative. These can be prescribed simultaneously with the launching of an exercise program. ¥ CT or MR angiography. For patients with inflow occlusive disease (weak femoral pulse), conventional catheter-based arteriography may be performed initially, in expectation of possible intervention. § Optimal candidates for peripheral bypass surgery have favorable anatomy for bypass (target vessel, good runoff, and, ideally, vein conduit), are medically fit, and have an anticipated life expectancy that will allow the patient to benefit from the procedure. ¶ Following revascularization, antiplatelet therapy depends upon the nature of revascularization (i.e., the type of stent, type of bypass conduit); if initiated, cilostazol may be stopped.

The treatment of PAD in patients with DM has two main goals: to improve peripheral blood flow in symptomatic patients and to treat vascular risk factors and concomitant disorders, with an emphasis on coronary and cerebrovascular vascular diseases. Exercise training, such as structured walking, should be prescribed, while weight control must be advocated for overweight diabetics. A step-by-step schematic approach is shown in Figure 2.

The American College of Cardiology/American Heart Association (ACC/AHA) 2016 guidelines [54] and the European Society of Cardiology/European Society for Vascular Surgery (ESC/ESVS) 2017 guidelines [55] strongly recommend smoking cessation, glycemic control, blood pressure control, and statin therapy. The European guidelines also recommend therapeutic targets: low-density lipoprotein (LDL) levels of less than 70 mg/dL or a >50% reduction from baseline value, with blood pressure < 140/90 mmHg. Both guidelines also suggest the use of inhibitors of the renin-angiotensin system to decrease ischemic events [54,55]. Metformin is the oral antidiabetic drug of choice in concomitant DM and PAD. An SGLT-2 inhibitor or GLP-1 agonist may also be used. More prudent SGLT-2 inhibitors are empagliflozin and dapagliflozin, as canagliflozin may increase the risk of amputation and should, therefore, be avoided. The use of basal insulin analogs is considered safe [56].

Both U.S. and European guidelines promote single antiplatelet therapy, either aspirin or clopidogrel, in symptomatic individuals. The U.S. guidelines recommend aspirin or clopidogrel to reduce coronary events [54], whereas the European guidelines do not support antiplatelet agents in asymptomatic cases unless other indications, such as coronary artery disease, are present [55]. Clopidogrel may be preferred to aspirin, according to the European guidelines, whereas the American guidelines refrain from making this statement. Dual antiplatelet therapy (DAPT) with aspirin and clopidogrel is only recommended for 1 month after percutaneous and surgical revascularization, according to the ESC guidelines (Class I) [55]. At the same time, the ACC guidelines give a Class-IIb recommendation for long-term dual antiplatelet inhibition [54]. The ACC guidelines also give anticoagulant therapy a Level-III harm recommendation and state that pentoxifylline is not effective for the treatment of claudication (also in Class III) [52]. Of note, the American guidelines promote the use of cilostazol (Class I, level of evidence A), whereas the European guidelines do not mention this drug. There is a strong emphasis on home-based training, either supervised or unsupervised (a Class-I recommendation in both guidelines).

Both guidelines have opted for a Class IIa-level recommendation for revascularization in patients with intermittent severe claudication. These procedures are recommended only when guideline-directed therapy and structured exercise programs fail. Although exercise therapy is crucial, its use is limited in frail patients or patients with ulcers or gangrene. The association between PAD and frailty syndrome in patients living with DM has recently been described [57]. Regarding endovascular approaches, both groups agree that revascularization should be performed in patients with hemodynamically significant upper inflow (aortoiliac) disease and lifestyle-limiting pain. For femoropopliteal disease (outflow vessels), the ACC guidelines advocate an endovascular approach, whereas the European guidelines favor such an approach only for femoropopliteal occlusions that are greater than 25 cm in length. However, both the ACC and ESC consent to surgical bypass for patients who are considered suitable for surgery. In Europe, the emphasis is on revascularization and contemporary therapies, whereas the American experts underline post-procedural surveillance and wound care [54,55]. The importance of surgical advice and consultation is, therefore, self-evident; an ideal multidisciplinary team for a diabetic patient with PAD would include an angiologist, a cardiologist, an interventionist, a vascular surgeon, a radiologist, and a diabetologist. The upcoming guidelines should emphasize this holistic approach, while the inclusion of a noninvasive protocol for assessment before and after revascularization would leverage the contribution of vascular imaging.

In cases of critical limb ischemia, both advisory bodies advocate revascularization. They also agree that endovascular and surgical revascularization procedures achieve similar outcomes. Controlling glycemia is a critical factor in reducing limb loss, foot wounds, and infection [54,55]. The relevance of DM is that both guidelines mention this practice and put forward the idea of educating these patients on self-foot examination and healthy foot behaviors.

Regarding acute limb ischemia (ALI), both guidelines support rapid emergency assessment and the initiation of heparin. If the limb is salvageable, the two groups again diverge: the American guidelines favor catheter-based thrombolysis (Class I) over percutaneous mechanical thrombectomy (Class IIa), surgical thromboembolectomy (Class IIa), and ultrasound-accelerated catheter-based thrombolysis (IIb) based on small studies, whereas the European guidelines do not recognize the superiority of thrombolysis over open surgical procedures [54,55]. Systemic thrombolysis has no role in ALI [58]. Catheter-based thrombolysis has a satisfactory clinical outcome, with a rate of 75–92% of complete or partial thrombus resolution, but patients with symptoms of longer duration (> 14 days) have better outcomes after surgery [58]. If blood flow is successfully restored after 24 h of continuous thrombolysis, angiography should be performed to identify preexisting arterial lesions that can be managed by endovascular (e.g., stenting) or surgical techniques (e.g., bypass).

Finally, it should be noted that at the time of publication of these guidelines, the results of the COMPASS [59] and VOYAGER PAD [60] trials were not published. Both studies have shown benefits in terms of cardiovascular events, including adverse limb events, compared with patients taking aspirin alone. The results of these studies, together with the introduction of SGLT2 inhibitors on a large scale, will bring new horizons and perhaps a dedicated section in future guidelines that will address the needs of this high-risk category of patients. Hypoglycemic drugs remain paramount but not sufficient; for example, a recent study showed that the drugs commonly used for the treatment of diabetes were inefficient at preventing the thickening of the basement membrane that is induced by the diabetogenic medium. Contrastingly, DAPT, a γ-secretase inhibitor that blocks the NOTCH pathway, stopped the thickening of the basement membrane in the organoids in vitro and in the diabetic mouse [61]. The mechanisms underlying diabetic vasculopathy are still not fully understood, which limits therapeutic drug development. As a matter of course, all cardiovascular disease risk factors associated with DM should be addressed: hypertension (BP < 130/80 mmHg), hyperlipidemia (LDL < 55 mg/dL), weight loss, physical activity, nutritional intake, smoking cessation, etc.

## 5. Conclusions

The therapeutic model of PAD in patients with DM is similar to the conventional treatment of nondiabetic patients. However, this strategy does not address the increased risk of cardiovascular events associated with DM, prompting future additional measures to target such a vulnerable group.

## Data Availability

We used PubMed and Web of Science to screen articles for this narrative review. We did not report any data.

## Abbreviations

PAD, peripheral artery disease; DM, diabetes mellitus; HbA1c, hemoglobin A1c; CRP, C-reactive protein; PKC, protein kinase C; LDL, low-density lipoprotein; ABI, ankle-brachial index; TBI, toe-brachial index; CT, computed tomography; MR, magnetic resonance; DAPT, dual antiplatelet therapy.
